# Effect of *Bacillus coagulans* SNZ 1969 on the Improvement of Bowel Movement in Loperamide-Treated SD Rats

**DOI:** 10.3390/nu14183710

**Published:** 2022-09-08

**Authors:** Soo-Min Jung, Ae-Wha Ha, Su-Jin Choi, Se-Young Kim, Woo-Kyoung Kim

**Affiliations:** 1 Department of Food Science and Nutrition, College of Science and Technology, Dankook University, Cheonan 31116, Korea; 2 R&D Center, CTCBIO, Inc., Hwaseong 18576, Korea; 3 Department of Agricultural Biotechnology, College of Agriculture and Life Sciences, Seoul National University, Seoul 08826, Korea

**Keywords:** *Bacillus coagulans*, probiotics, constipation, bowel movement, mucin, gastrointestinal hormone

## Abstract

*Bacillus coagulans* SNZ 1969 (*B. coagulans* SNZ 1969) is a spore-forming bacterium reported to be effective in attenuating constipation. However, there is no study on whether *B. coagulans* SNZ 1969 could improve constipation through mucin secretion and changes in intestinal hormones. To address this knowledge gap, rats were orally administrated with various treatments for four weeks. The normal control (NOR) group received saline only. There were four constipation-induced groups. The LOP group received only loperamide (LOP), a constipation-inducing agent. The BIS group received both LOP and Bisacodyl (BIS, a constipation treatment agent). The SNZ-L group received both LOP and *B. coagulans* SNZ 1969 at 1 × 10^8^ CFU/day. The SNZ-H group received LOP and *B.* *coagulans* SNZ 1969 at 1 × 10^10^ CFU/day. As indicators of constipation improvement, fecal pellet weight, fecal water content, gastrointestinal transit time, and intestinal motility were measured. Mucus secretion in the colon was determined by histological colon analysis and mucin-related gene expressions. Gastrointestinal (GI) hormones were also measured. SNZ-L and SNZ-H groups showed significantly increased fecal weights, fecal water contents, and intestinal motility than the LOP group. SNZ-L and SNZ-H groups also showed higher secretion of mucin in the colon and mRNA expression levels of Mucin 2 and Aquaporin 8 than the LOP group. The SNZ-H group showed significantly increased substance P but significantly decreased somatostatin and vasoactive intestinal peptide than the LOP group. The results of this study suggest that *B. coagulans* SNZ 1969 intake could attenuate constipation through mucin secretion and alteration of GI hormones.

## 1. Introduction

Constipation is a symptom in which bowel movements are less frequent (usually fewer than three times a week), and stools are hard, making it difficult to pass through the intestine [1]. In modern society, the number of people with constipation symptoms is increasing due to various environmental, genetic, social, and economic reasons [2]. Constipation can be alleviated by increasing the intake of sufficient fiber. However, dietary fiber absorbs water in the large intestine and increases the volume of the abdomen, causing discomfort such as bloating [3,4]. Long-term use of constipation medications such as osmotic laxatives or stimulant laxatives may cause abdominal distension and diarrhea with various complications [5].

As a functional food, probiotics have recently received attention for gut health [5]. Probiotics are defined as “living microorganisms that, when administered in appropriate doses, confer a health benefit on the host’’ [6]. The microorganisms mostly used as probiotics belong to the families of *Lactobacillus* and *Bifidobacterium* [7]. Among the Bacillus species, *Bacillus coagulans* SNZ 1969 is a lactic acid-producing bacteria. It is a rod-shaped, weakly acidic, gram-positive, and spore-forming bacterium [8]. Spore-forming Bacillus species have the advantage of surviving in the intestine and passing through the intestinal tract due to their resistance to high heat and acidic conditions [9]. *B. coagulans* SNZ 1969 was originally isolated in Japan in 1949 from green malt [10]. *B. coagulans* is also an economically important species that is frequently involved in the production of lactic acid, coagulin, and other thermostable enzymes [11]. *B. coagulans* has been stated as safe for human consumption by the US Food and Drug Administration (FDA) and the European Union Food Safety Authority (EFSA). It is on the list of Generally Recognized As Safe (GRAS) and Qualified Presumption of Safety (QPS) [12,13].

Potential mechanisms of *B. coagulans* for attenuating constipation include changes in gut microbiota, decreased pH due to increased production of short-chain fatty acids, and improved intestine motility [14,15,16,17,18,19,20,21,22]. However, as a mechanism for preventing constipation by *B. coagulans* SNZ 1969, it is not known whether intestinal motility is related to alterations of mucin secretion and/or GI hormones. Therefore, the purpose of this study was to determine how *B. coagulans* SNZ 1969 could improve intestinal motility and its action mechanism in an animal model.

## 2. Materials and Methods

### 2.1. Experimental Design

Forty-eight five-week-old male Sprague Dawley rats were purchased from Dooyeol biotech (Seoul, Korea). These experimental animals were fed AIN-93G (Dooyeol biotech, Seoul, Korea) and acclimatized to the laboratory environment for one week. Each rat was housed in a separate clean cage with a temperature of 22 ± 1 °C, a humidity of 60 ± 5%, and a light/dark cycle of 12 h/12 h. After one week, the body weight of each rat was measured. Based on their body weights, rats were classified into five groups (eight animals in each group) by a randomized block design.

Experimental groups included one normal control group (NOR) and four constipation groups induced by loperamide (LOP), including: (1) LOP group (receiving only loperamide), (2) BIS group (receiving LOP + Bisacodyl (BIS, the constipation treatment agent)), (3) SNZ-L group (receiving LOP + *B.coagulans* SNZ 1969 at 1 × 10^8^ CFU/day), and (4) SNZ-H group (receiving LOP + *B.coagulans* SNZ 1969 at 1 × 10^10^ CFU/day).

**Figure 1:**
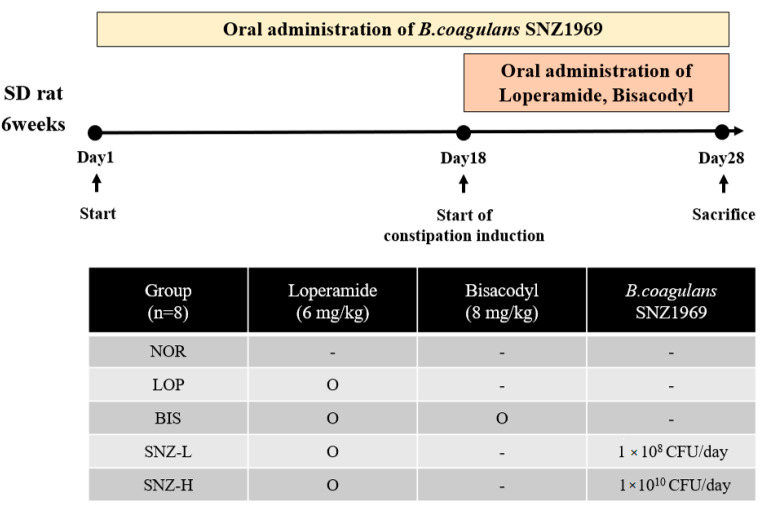
Experimental design. Experimental animals were five-week-old male SD rats. NOR: Normal group; LOP: Loperamide treated group; BIS: Loperamide and Bisacodyl 8 mg/kg treated group; SNZ-L: Loperamide and *B. coagulans* SNZ 1969 at 1 × 10^8^ CFU/day treated group; SNZ-H: Loperamide and *B. coagulans* SNZ 1969 at 1 × 10^10^ CFU/day treated group.

The experimental process is shown in Figure 1. *B. coagulans* 1969 was dissolved in 1 mL of saline and orally administered to both SNZ-L and SNZ-H groups once a day for 4 weeks, respectively. NOR, LOP, and BIS groups were orally administered with 1 mL of physiological saline to create the same conditions as the SNZ groups. On the 18th day of breeding, 6 mg/kg of loperamide (Sigma-Aldrich Co., St. Louis, MO, USA) was orally administered to all groups (except for the NOR group) to induce constipation. After 30 min of loperamide administration, physiological saline was orally administered to the LOP group. BIS group was orally administered with bisacodyl (Sigma-Aldrich Co., St. Louis, MO, USA) at a dose of 8 mg/kg for 11 days. Food and water were provided *ad libitum*. The total experimental period was four weeks. The experiment was conducted after obtaining approval from the Experimental Animal Steering Committee of Dankook University (DKU-21-054).

### 2.2. Measurements of Body Weight, Food Intake, and Dietary Efficiency Ratio

The body weight of each experimental animal was measured once a week at a fixed time. The weight gain was calculated as the difference between the final body weight to the initial body weight of the experimental animal. The food intake was measured twice a week at a fixed time, calculated as the difference between the food supply and the remaining amount. The food efficiency ratio (FER) was calculated by dividing the weight gain during the four weeks by the total food intake during the same period.

### 2.3. Collection of Samples

After experimental animals fasted for 24 h, 1 mL (60 mg/mL in 0.9% NaCl) of carmine red (Sigma-Aldrich Co., St. Louis, MO, USA) was orally administered to all groups to measure intestinal mobility. After 30 min, animals were anesthetized using a CO_2_ chamber and sacrificed. After performing a laparotomy for experimental animals, blood was collected from the abdominal aorta. The collected blood was placed in a tube containing heparin (Bacton Dickinson, Franklin Lakes, NJ, USA) and centrifuged (Gyrozen, Deajeon, Korea) at 3000 rpm for 15 min at 4 °C. The plasma was then collected and stored in a −70 °C deep freezer for later analysis. After blood collection, the small intestine was collected to measure intestinal motility, and the large intestine was used for histological analysis. The liver, kidney, epididymal fat, spleen, and thymus were removed. The blood around each organ was removed using 0.9% NaCl solution (JW-pharma, Seoul, Korea). The weights of organs were then measured.

### 2.4. Measurement of Fecal Pellet Weight and Water Content

On the 10th day of loperamide administration, fecal pellets were collected for 24 h and weighed. The fecal pellet was dried in a 70 °C dry oven until the weight of the fecal pellet was constant. The fecal water content was calculated based on the difference between wet and dry fecal weights as follows:

Fecal water content (%) = 100 (wet weight − dry weight)/wet weight

### 2.5. Measurement of Gastrointestinal Transit Time

On the fourth day of induction of constipation, experimental animals were fasted for 24 h, and then an AIN-93G diet mixed with 0.5% carmine red (Sigma-Aldrich Co., St. Louis, MO, USA), a marker for intestinal transit, was fed. After feeding the carmine red mixed diet, whether red marks appeared in the stool at 30-min intervals was checked. The difference between the start time of the carmine red mixed diet fed and the time when the first red stool appeared was defined as the intestinal transit time.

### 2.6. Measurement of Intestinal Motility

To measure intestinal motility, animals were fasted for 24 h before the end of the experiment. At the end of the experiment, 1 mL (60 mg/mL in 0.9% NaCl) of carmine red (Sigma-Aldrich Co., St. Louis, MO, USA) was orally administered 30 min after loperamide treatment. After 30 min, the experimental animals were sacrificed, gastrointestinal tracts were removed, lengths of small intestines were measured, and lengths of movement of carmine red from the stomach were measured. Intestinal motility (%) was calculated by dividing the length of movement of carmine red from the stomach by the total length of the small intestine (the length of the stomach and ileum) using the following formula:

Intestinal motility (%) = distance traveled by carmine red/total length of the small intestine × 100

### 2.7. Measurement of Mucus Secretion in the Colon

(1) Histological analysis.

The colon tissue was fixed in 10% neutral buffered formalin solution (Sigma-Aldrich Co., St. Louis, MO, USA) after dissecting the lumen and spreading to expose the mucosal surface. The fixed tissue was embedded in paraffin according to the general tissue processing method. The paraffin block was cut into sections with a thickness of about 4 μm using a thin slicer. Sections were stained with Alcian blue stain (pH 2.5) (Abcam, Cambridge, UK) and examined under an optical microscope (Olympus BX53, Tokyo, Japan) at X200 magnification.

(2) mRNA expression levels of Mucin 2 and Aquaporin 8.

For RNA isolation and purification, the colon tissue stored at −70 °C was homogenized, and total mRNA was extracted using TRI reagent (Sigma-Aldrich Co., St. Louis, MO, USA). mRNA expression levels of Mucin 2 and Aquaporin 8 were measured by real-time polymerase chain reaction (RT-PCR) with the same method mentioned in the previous study by the same authors [23]. Sequences of primers used in this study are listed in Table 1. Glyceraldehydes-3-phosphate dehydrogenase (GAPDH) was used as a control indicator.

### 2.8. Gastrointestinal Hormones

Gastrointestinal hormones such as gastrin (Gas), substance P (SP), cholecystokinin (CCK), somatostatin (SS), and vasoactive intestinal peptide (VIP) in plasma were measured using enzyme-linked immunosorbent assay (ELISA) kits (Cusabio, Houston, TX, USA) according to the manufacturer’s instructions.

### 2.9. Statistical Analysis

All results of this experiment were analyzed using the IBM SPSS Statistics 26.0 (SPSS Inc., Chicago, IL, USA) program. All values are expressed as mean ± standard error. Duncan’s multiple range test was performed to verify the significance of differences between groups at the significance level of *p* < 0.05 after analysis of variance (ANOVA).

## 3. Results

### 3.1. Body Weight, Dietary Intake, and Food Efficiency Ratio (FER)

The results of body weight, dietary intake, and dietary efficiency of experimental animals are shown in Table 2. There was no significant difference in the initial body weight among groups (*p* = 0.884). However, the final body weight was significantly (*p* < 0.05) decreased in the BIS group compared to that in the NOR group. Both the LOP group and SNZ-treated group showed a tendency to decrease in final weight compared to the NOR group, although the decrease was not statistically significant (*p =* 0.071). There was no significant difference in dietary intake (*p* = 0.442) or FER (*p* = 0.281) among groups.

### 3.2. Various Organ Weights of Experimental Groups

The weights of liver and epididymal fat were significantly lower in the BIS group than in the NOR group (Table 3, *p* < 0.05). There was no significant difference in the weight of the kidney (*p* = 0.323), spleen (*p* = 0.609), or thymus (*p* = 0.594) among groups. *B. coagulans* SNZ 1969 treated groups only showed a statistical difference in liver weight compared to the NOR group. However, they showed no statistical significance in all organ weights compared to the LOP group or the BIS group (*p* > 0.05).

### 3.3. Fecal Pellet Weight and Fecal Water Content

**Figure 2:**
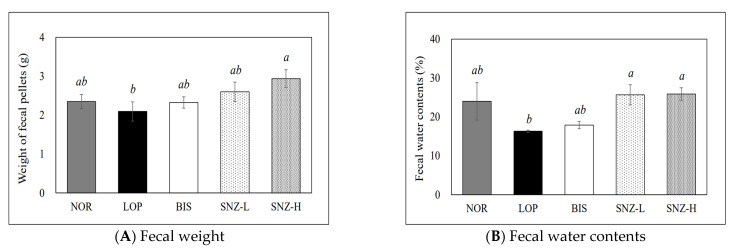
Effects of *B. coagulans* SNZ 1969 on fecal weight (**A**) and fecal water contents (**B**) in loperamide-induced constipation rats. NOR: Normal group; LOP: Loperamide treated group; BIS: Loperamide and Bisacodyl 8 mg/kg treated group; SNZ-L: Loperamide and *B. coagulans* SNZ 1969 at 1 × 10^8^ CFU/day treated group; SNZ-H: Loperamide and *B. coagulans* SNZ 1969 at 1 × 10^10^ CFU/day treated group. Results are presented as mean ± SE (n = 8). Means with the different letters above a bar are significantly different at *p* < 0.05 by Duncan’s multiple range test.

Results of fecal weights and fecal water contents of experimental animals induced with loperamide are shown in Figure 2. Fecal weight showed a tendency to decrease in the LOP group with a tendency to increase in the BIS group and SNZ groups compared to that in the LOP group. The SNZ-H group had the highest fecal weight among all other experimental groups. The SNZ-H group and LOP group showed a significant difference in fecal weight (*p* < 0.05). The results of fecal water content were similar to those of fecal weight. The fecal water content showed a tendency to decrease in the LOP group and a tendency to increase in the BIS group and SNZ groups. Fecal water contents in both SNZ-L and SNZ-H groups were significantly (*p* < 0.05) increased compared to those of the LOP group (Figure 2B).

### 3.4. Gastrointestinal Transit Time and Intestinal Motility

#### 3.4.1. Gastrointestinal Transit Time

**Figure 3:**
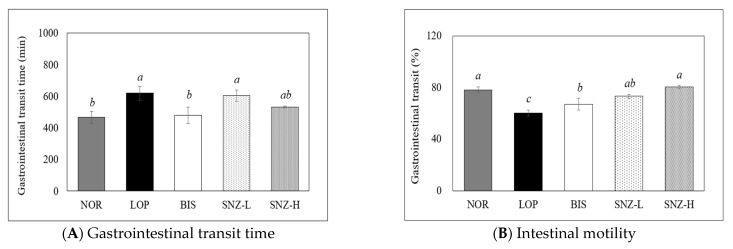
Effects of *B. coagulans* SNZ 1969 on (**A**) gastrointestinal transit time and (**B**) intestinal motility. NOR: Normal group; LOP: Loperamide treated group; BIS: Loperamide and Bisacodyl 8 mg/kg treated group; SNZ-L: Loperamide and *B. coagulans* SNZ 1969 at 1 × 10^8^ CFU/day treated group; SNZ-H: Loperamide and *B. coagulans* SNZ 1969 at 1 × 10^10^ CFU/day treated group. Results are presented as Mean ± SE (n = 8). Means with the different letters above a bar are significantly different at *p* < 0.05 by Duncan’s multiple range test.

The intestinal transit time was defined as the difference between the start time of the carmine red mixed diet fed and the time when the first red stool appeared. The results of intestinal transit time on the fourth day after administration of loperamide are shown in Figure 3a. The intestinal transit time was significantly increased in the LOP group compared to that in the NOR group. The BIS group showed a significantly decreased intestinal transit time compared to the LOP group (*p* < 0.05). In the case of the SNZ-H group, the intestinal transit time showed a tendency to decrease compared to the LOP group, although the decrease was not statistically significant (*p* > 0.05).

#### 3.4.2. Intestinal Motility

The movement distance of the dyeing reagent (as intestinal motility (%)) was confirmed in the small intestine (Figure 3B). The intestinal motility in the LOP group was significantly decreased compared to that in the NOR group. The intestinal motility in the BIS group showed a tendency to increase compared to that in the LOP group, although such an increase was statistically insignificant (*p* > 0.05). The group administered with *B. coagulans* SNZ 1969 showed significantly increased intestinal motility compared to the LOP group (*p* < 0.05). In the case of the SNZ-H group, intestinal motility was significantly increased as much as that in the NOR group.

### 3.5. Secretion of Mucus in the Large Intestine

#### 3.5.1. Histological Analysis

**Figure 4:**
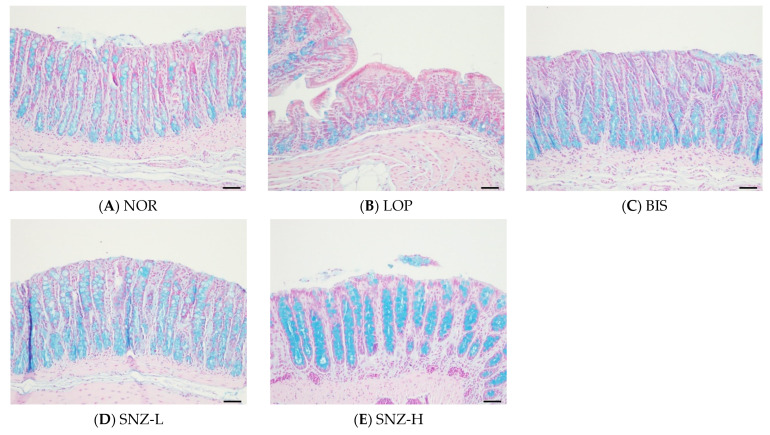
Effects of *B. coagulans* SNZ 1969 on mucous secretion capacity in loperamide-induced constipation rats. (**A**) NOR: Normal group, (**B**) LOP: Loperamide treated group, (**C**) BIS: Loperamide and Bisacodyl 8 mg/kg treated group, (**D**) SNZ-L: Loperamide and *B. coagulans* SNZ 1969 at 1 × 10^8^ CFU/day treated group, (**E**) SNZ-H: Loperamide and *B. coagulans* SNZ 1969 at 1 × 10^10^ CFU/day treated group. Magnification 200×. Scale bar = 50 μm.

The results of the histological evaluation of the colon are shown in Figure 4. The mucinous substance of the colon is shown in blue when stained in the NOR group. The level of mucinous substance was decreased in the LOP group. On the other hand, in BIS and the SNZ-treated groups, similar levels of mucinous substances as in the NOR group were observed. In particular, the level of mucinous substances was significantly prominent in the SNZ-H group.

#### 3.5.2. mRNA Expression of Mucin 2 and Aquaporin 8

**Figure 5:**
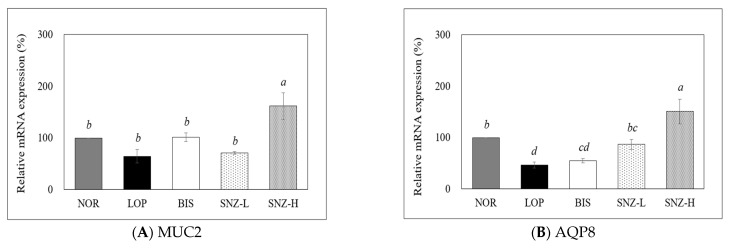
Effects of *B. coagulans* SNZ 1969 on mRNA expression of MUC2 (**A**) and AQP8 (**B**) in the transverse colon of loperamide-induced constipation rats. NOR: Normal group; LOP: Loperamide treated group; BIS: Loperamide and Bisacodyl 8 mg/kg treated group; SNZ-L: Loperamide and *B. coagulans* SNZ 1969 at 1 × 10^8^ CFU/day treated group; SNZ-H: Loperamide and *B. coagulans* SNZ 1969 at 1 × 10^10^ CFU/day treated group. Total mRNA was isolated by Tri-reagent and used for cDNA synthesis and real-time PCR. The GAPDH level was used for comparison as a loading control. The results were analyzed by the ∆∆CT method, and statistical treatment was performed by repeating three times independently. The results are presented as mean ± SE (n = 8). Means with different letters above a bar are significantly different at *p* < 0.05 by Duncan’s multiple range test.

There were significant differences in the mRNA expression of Mucin 2 (MUC2) among groups (NOR, LOP, BIS, and SNZ-L groups). In the SNZ-H group, the mRNA expression of MUC2 was significantly increased compared to those in the other groups (Figure 5A) (all *p* < 0.05). The mRNA expression of Aquaporin 8 (AQP8) was significantly decreased in the LOP group compared to that in the NOR group but significantly increased in both SNZ-L and SNZ-H groups compared to that in the LOP group (Figure 5B) (all *p* < 0.05).

### 3.6. Measurement of Gastrointestinal Hormones

**Figure 6:**
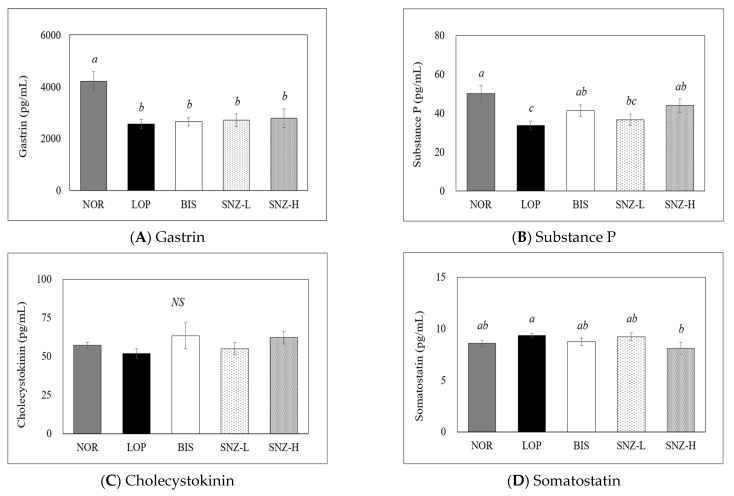

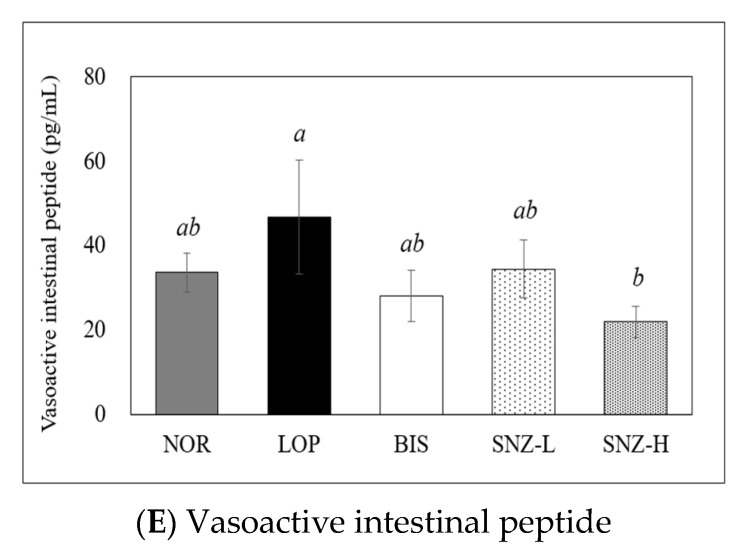
Effects of *B. coagulans* SNZ 1969 on plasma gastrointestinal hormones in loperamide-induced constipation rats. NOR: Normal group, LOP: Loperamide treated group; BIS: Loperamide and Bisacodyl 8 mg/kg treated group; SNZ-L: Loperamide and *B. coagulans* SNZ 1969 at 1 × 10^8^ CFU/day treated group; SNZ-H: Loperamide and *B. coagulans* SNZ 1969 at 1 × 10^10^ CFU/day treated group. Concentrations of (**A**) Gastrin, (**B**) Substance P, (**C**) Cholecystokinin, (**D**) Somatostatin, and (**E**) Vasoactive intestinal peptide in plasma were estimated by ELISA. The results are presented as mean ± SE (n = 8). Means with the different letters above a bar are significantly different at *p* < 0.05 by Duncan’s multiple range test. NS: not significant.

Concentrations of gastrin (Gas), substance P (SP), cholecystokinin (CCK), somatostatin (SS), and vasoactive intestinal peptide (VIP) in plasma samples of experimental animals are shown in Figure 6. The Gas level was significantly (*p* < 0.05) decreased in groups treated either with loperamide alone or loperamide with BIS or loperamide with SNZ treatment compared to that in the NOR group (Figure 6A). The SP level was significantly decreased in the LOP group compared to that in the NOR group but was significantly increased in the BIS group and the SNZ-H group compared to that in the LOP group (Figure 6B) (all *p* < 0.05). There was no significant difference in CCK concentration among groups (*p* = 0.415) (Figure 6C). The concentration of SS showed a significant decrease only in the SNZ-H group compared to that in the LOP group (Figure 6D) (*p* < 0.05). VIP concentrations in the BIS group, SNZ-L group, and SNZ-H group also tended to decrease compared to that in the LOP group, but statistical significance was found only in the LOP group and SNZ-H group (*p* < 0.05) (Figure 6E).

## 4. Discussion

One of the symptoms of constipation is the hardening of the stool due to a decrease in water in the stool. A study on the correlation between decreased stool weight caused by constipation and the water content of the stool has been reported [1]. Thus, fecal weight and fecal water content were measured after two levels of *B. coagulans* SNZ 1969 were administered to constipated rats. In this study, fecal weight and fecal water content were significantly decreased in the LOP group compared to the NOR group, confirming that constipation was induced due to loperamide. Meanwhile, when *B. coagulans* SNZ 1969 was ingested in the state of induced constipation, the fecal weight and fecal water content were significantly increased compared to those in the LOP group, confirming that *B. coagulans* SNZ 1969 was effective in attenuating constipation.

Proper concentrations of probiotics used in constipation-induced rats were 10^6^–10^10^ colony-forming units (CFU) in different studies [14,15,16,17,23,24,25]. Accordingly, in this study, 10^8^ as a low concentration and 10^10^ as a high concentration of B. coagulans SNZ 1969 were orally administrated to constipation-induced rats. In this study, the high concentration of B. coagulans SNZ 1969 (1 × 10^10^ CFU) intake had a greater effect in attenuating constipation and intestinal motility than the low concentration. Previous studies have shown no death or toxicity symptoms when animal models receive the highest dose level of 1.36 × 10^11^ CFU *B. coagulans* kg BW/day [24,25].

This study also measured intestinal transit time and intestinal motility. Together with the above results, they could determine the interlocking effect of the large intestine and small intestine on constipation. The intestinal transit time was measured as the total motility of the gastrointestinal tract (including the small intestine and large intestine) by the time for the red marker to first appear in the stool after feeding the carmine red mixed diet. On the other hand, the intestinal mobility rate measures the degree of movement in the small intestine. This study showed that feeding *B. coagulans* SNZ 1969 in constipated rats shortened the passage time of stool in the large intestine compared to the LOP group, although the difference was not statistically significant. Meanwhile, the intestinal motility decreased by loperamide was increased significantly by SNZ in a dose-dependent manner.

Loperamide is used as an agent to set a constipated animal model. It could induce constipation by inhibiting intestinal water secretion or by inhibiting intestinal smooth muscle contraction, thus reducing bowel movements [26,27]. Therefore, contrary to the mechanism of action of loperamide, the ingestion of *B. coagulans* SNZ 1969 improved constipation by activating intestinal motility. During the experiment period, diet intake and weight gain between SNZ groups and other experimental groups were not statistically different, meaning that the improvement of constipation in the SNZ groups resulted from the intake of *B. coagulans* SNZ1969. This result is consistent with previous studies showing that various strains of probiotics are effective at preventing constipation [14,15,16,17,18,19,20]. Various strains of *B. coagulans* can improve the imbalance of colonic microflora by enhancing beneficial bacteria, *Lactobacillus*, reducing the harmful bacteria *Clostridium,* and further increasing the peristalsis of the colon, thereby attenuating constipation [15,16,17]. Some clinical studies have also confirmed that the intake of *B. coagulans* SNZ 1969 can improve intestinal motility and microbial alterations [18]. *B. coagulans* can produce lactic acid and short-chain fatty acids, which can reduce the pH of the colon, strengthen peristalsis, reduce colonic transit time, and further improve defecation frequency, fecal volume, and fecal characteristics [19,20,21]. Another clinical study by Maity et al. [28] also reported that the intake of *B. coagulans* LBSC can improve intestinal microbiota in irritable bowel syndrome subjects.

Next, this study investigated whether the constipation improvement effect of *B. coagulans* SNZ 1969 was related to changes in mucin secretion and GI hormones known to affect intestinal motility. In histological examination, the group with *B. coagulans* treatment showed more mucin secretion in the colon of rats compared to the LOP group. Consistent with those results, expression levels of mucin-related genes MUC2 and AQP8 were also increased after *B. coagulans* treatment. Meanwhile, all mucin-related indices were decreased in the LOP group. The mucus can lubricate fecal flow, making it easier to defecate [29]. Studies have reported that a decreased thickness of the mucus in the large intestine can interfere with intestinal motility when loperamide is administered to rats, which can result in worsened constipation [30,31]. A clinical study on chronic constipation has also reported that the supplementation of a mucus stimulant can result in profoundly increased mucin and viscosity, which, in turn, can accelerate gastrointestinal transit and evacuation of non-digestible food components in feces [32]. Consistent with the studies described above, this study also confirmed that the intake of *B. coagulans* SNZ 1969 could relieve constipation symptoms by increasing mucin secretion, maintaining intestinal lubricity, and further increasing intestinal motility.

In this study, among the five gastrointestinal (GI) hormones, Substance P (SP) and an indicator of gastrointestinal peristalsis were significantly increased, whereas somatostatin (SS) and Vasoactive intestinal peptide (VIP) levels were decreased after treatment with a high concentration of *B. coagulans* SNZ 1969 compared to loperamide treatment. SP is an excitatory peptide neurotransmitter that can stimulate bowel movements and relieve constipation. However, SS and VIP are inhibitory peptide neurotransmitters that can inhibit the contraction of the gastrointestinal tract and intestinal motility [33]. Studies have reported that GI hormones such as motilin (MTL), cholecystokinin (CCK), gastrin (Gas), and SP are significantly decreased while SS and VIP are increased in constipation-induced rats [34,35,36,37]. Altered GI hormones in slow colonic transit constipation patients play important roles in the regulation of GI motility [38]. A study on treatment with *B. adolescentis*, a probiotic, significantly increased levels of MTL, Gas, and SP but decreased levels of SS and VIP compared with constipation-induced rats, which stimulated peristalsis and transport of feces, thus attenuating constipation [37]. The result of this study was consistent with the above studies, suggesting that changes in GI hormones, especially SP, SS, and VIP, after supplementation of *B. coagulans* SNZ 1969 relieved constipation by enhancing intestinal motility. One thing that needs to be considered is that among the five hormones investigated in this study, only three showed statistical significance between probiotic treatment and loperamide treatment. Thus, whether the change of GI hormones is an important factor for constipation improvement by *B. coagulans* SNZ 1969 remains to be clarified.

## 5. Conclusions

In this study, the effect of *B. coagulans* SNZ 1969 administration in attenuating intestinal function was evaluated in an animal model of loperamide-induced constipation. *B. coagulans* SNZ 1969 increased fecal weights and fecal water contents, ultimately increasing intestinal motility. Furthermore, the present study suggests that *B. coagulans* SNZ 1969 can enhance intestinal motility by regulating mucin secretion and GI hormones such as SP, SS, and VIP. The results of this study suggest that *B. coagulans* SNZ 1969 intake might be effective in attenuating constipation by enhancing mucin secretion and GI hormone alterations.

## Figures and Tables

**Table 1:** Primer sequences of transcription factors used for real-time PCR analysis.

Gene ^(1)^	Primer	Sequence ^(2)^
GAPDH	Forward primer	5’-AGTGCCAGCCTCGTCTCATA-3’
	Reverse primer	5’-ACCAGCTTCCCATTCTCAGC-3’
MUC2	Forward primer	5’-GGTGGCCTTCAAATCAGGTG-3’
	Reverse primer	5’-AGGGTTTGAAGATGGAGAAGCTC-3’
AQP8	Forward primer	5’-CAGATATGTCTGGGGAGCAG-3’
	Reverse primer	5’-GCCTAATGAGCAGTCCCACA-3’

^(1)^ GAPDH: Glyceraldehydes-3-phosphate dehydrogenase; MUC2: mucin 2; AQP8: aquaporin 8. ^(2)^ T: Thymine; A: Adenine; C: Cytosine; G: Guanine.

**Table 2:** Initial and final body weight, total dietary intakes, and food efficiency ratio (FER).

Group	Initial Weight (g)	Final Weight (g)	Weight Gain (g/28 days)	Diet Intake (g/28 days)	FER ^(1)^
NOR ^(2)^	195.6 ± 5.9 ^NS,(3)^	354.2 ± 5.0 ^(4),a,(5)^	158.6 ± 7.6 ^NS^	469.8 ± 11.3 ^NS^	0.34 ± 0.01 ^NS^
LOP	197.8 ± 4.9	342.8 ± 6.7 ^a,b^	145.1 ± 6.9	453.5 ± 13.4	0.32 ± 0.01
BIS	192.4 ± 11.4	329.1 ± 4.5 ^b^	136.7 ± 11.5	448.2 ± 10.1	0.30 ± 0.02
SNZ-L	198.4 ± 4.0	347.3 ± 5.0 ^a^	148.9 ± 5.5	465.3 ± 9.4	0.32 ± 0.01
SNZ-H	202.0 ± 3.9	348.7 ± 3.9 ^a^	146.7 ± 5.1	473.9 ± 11.0	0.31 ± 0.00

^(1)^ FER: Body weight gain for 28 days (g)/food intake for 28 days (g). ^(2)^ NOR; Normal group, LOP; Loperamide treated group, BIS; Loperamide and Bisacodyl 8 mg/kg treated group, SNZ-L; Loperamide and *B. coagulans* SNZ 1969 at 1 × 10^8^ CFU/day treated group, SNZ-H; Loperamide and *B. coagulans* SNZ 1969 at 1 × 10^10^ CFU/day treated group. ^(3), NS^: Not significant. ^(4)^ Mean ± SE (n = 8). ^(5)^ Means with different letters within each row are significantly different at *p* < 0.05 by Duncan’s multiple range test.

**Table 3:** Weights of organs of experimental groups (g).

Group	Liver	Kidney	Epididymal Fat Pad	Spleen	Thymus
NOR ^(1)^	10.55 ± 0.33 ^(2),a,(3)^	2.66 ± 0.08 ^NS,(4)^	5.27 ± 0.287 ^a^	0.72 ± 0.02 ^NS^	1.61 ± 0.09 ^NS^
LOP	9.90 ± 0.27 ^a,b^	2.57 ± 0.08	5.29 ± 0.46 ^a^	0.68 ± 0.01	1.67 ± 0.10
BIS	9.28 ± 0.32 ^b^	2.51 ± 0.06	3.93 ± 0.26 ^b^	0.70 ± 0.03	1.59 ± 0.06
SNZ-L	9.33 ± 0.24 ^b^	2.58 ± 0.07	4.65 ± 0.29 ^a,b^	0.70 ± 0.04	1.70 ± 0.09
SNZ-H	9.31 ± 0.37 ^b^	2.45 ± 0.06	4.75 ± 0.51 ^a,b^	0.74 ± 0.03	1.76 ± 0.06

^(1)^ NOR: Normal group; LOP: Loperamide treated group; BIS: Loperamide and Bisacodyl 8 mg/kg treated group; SNZ-L: Loperamide and *B. coagulans* SNZ 1969 1 × 10^8^ CFU/day treated group; SNZ-H: Loperamide and *B. coagulans* SNZ 1969 1 × 10^10^ CFU/day treated group. ^(2)^ Mean ± SE (n = 8). ^(3)^ Means with different letters within each row are significantly different at *p* < 0.05 by Duncan’s multiple range test. ^(4), NS^: Not significant.
